# The Dynamics of Changes and Spatial Differences in the Synthetic Indicator for Evaluating Environmental Performance in Poland: Current State

**DOI:** 10.3390/ijerph16224490

**Published:** 2019-11-14

**Authors:** Adam Senetra, Katarzyna Pawlewicz, Adam Pawlewicz

**Affiliations:** 1 Institute of Geography and Land Management, University of Warmia and Mazury in Olsztyn, Prawocheńskiego Str. 15, 10-720 Olsztyn, Poland; adam.senetra@uwm.edu.pl; 2 Department of Agrotechnology, Agricultural Production Management and Agribusiness, University of Warmia and Mazury in Olsztyn, Oczapowskiego Str. 8, 10-719 Olsztyn, Poland; adampawl@uwm.edu.pl

**Keywords:** environmental indicators, environmental performance, synthetic indicator, multivariate comparative analysis, funding for environmental projects, quality of the natural environment, TOPSIS method, Poland

## Abstract

Socioeconomic development and consumption are among the key drivers of environmental degradation. Legal measures and the appropriate funding are required to effectively protect the natural environment. The aim of this study was to analyze the dynamics of changes and spatial differences in the measures undertaken to protect and improve the quality of the environment. A set of indicators for evaluating environmental performance was developed and tested on Poland as an example. The relevant data are publicly available in statistical databases. Proposed indicators can be modified for use in other countries by incorporating country-specific characteristics. The environmental protection activities implemented in Polish voivodeships at the Nomenclature of Territorial Units for Statistics (NUTS) 4 level (counties) in three financial frameworks (2004–2006, 2007–2013 and 2014–2017) were analyzed against the base year (2003). A total of 27 variables divided into four categories were analyzed: (1) water and wastewater management and water conservation, (2) waste management and protection of the Earth’s surface, (3) air pollution and climate control, (4) nature conservation and promotion of pro-environmental behaviors. A Synthetic Indicator for Evaluating Environmental Performance (SIEEP) was developed based on the Technique for Order Preference by Similarity to Ideal Solution (TOPSIS) method. Based on the arithmetic mean and standard deviation, the analyzed counties were divided into four typological classes reflecting the values of the SIEEP. The research showed that the implementation of environmental protection measures financed from public funds minimizes the negative impact of human activities on the environment. Positive changes in the values of the analyzed variables and a steady increase in the number of counties with high values of the SIEEP testify to the above.

## 1. Introduction

Socioeconomic growth, increasing consumption, and low levels of environmental awareness maximize the negative impact of human activities on the environment. To minimize these risks, governments introduce laws that influence the decisions made by both businesses and households. Considerable public funds are also channeled to environmental protection projects. To effectively mitigate the negative consequences of environmental degradation, cooperation is required not only at the national level, but also between governments and international institutions at the global level [1]. The right to live in a safe and healthy environment is a fundamental human right [2]. The consumption of energy generated from various sources leads to growing levels of pollution. This global problem poses a considerable threat to human health and to life. Industrial, municipal, and agricultural pollution compromises the quality of terrestrial and aquatic ecosystems on a global scale [3,4]. Consequently, numerous attempts are made to examine the effects of social factors on the environment [5,6,7].

Many Polish regions are characterized by unspoiled nature, traditional rural landscapes, and a relatively small proportion of degraded soils. For this reason, direct funding for environmental projects had been limited in the past. The significance of environmental pollution was recognized only in the early 1990s. Similar processes could be observed in other Eastern Bloc countries which are now European Union members. In most of these countries, the state of the natural environment was catastrophic after 40 years of communist rule [8]. The expert reports developed at that time revealed that Poland, East Germany (the former German Democratic Republic), and Czechoslovakia (Czech Republic and Slovakia since 1 January 1993) were the most polluted countries in Europe, mainly due to irrational decision-making in their centrally planned economies [9,10]. The Soviet economic model led to high levels of air and water pollution, as well as considerable soil degradation [11,12]. The importance of rational resource management and environmental protection was recognized at the beginning of the political transformation process, and these issues became the priority goals on Poland’s road to development [13]. In the past thirty years, considerable progress has been made in minimizing the environmental impact of human activities. Despite the above, further measures are needed to improve the eco-efficiency of the Polish economy [14].

Environmental protection efforts and programs gained speed when Poland joined the European Union (EU) in 2004 and became eligible for EU funding. The expenditure on fixed assets in environmental protection increased. Three financial frameworks for environmental grants can be identified since the beginning of Poland’s membership in the EU [15,16,17]:

The first financial framework (2004–2006), during which environmental programmes received support under the European Union’s Cohesion Policy for the 2000–2006 programming period. Grants for environmental projects were also obtained under the Integrated Regional Operational Programme (IROP) 2004–2006 and the Improvement of the Competitiveness of Enterprises Sectoral Operational Programme 2004–2006 (ICE SOP) of the European Regional Development Fund (ERDF). Funding for environmental initiatives was also provided by the Restructuring and Modernization of the Food Sector and Rural Development Sectoral Operational Programme in the 2004–2006 period (Agri SOP) of the European Agricultural Guidance and Guarantee Funds (EAGGF).

The second financial framework (2007–2013), during which environmental programmes received support under the Infrastructure and Environment Operational Programme in the 2007–2013 period (I&E OP) of the Cohesion Fund (CF), and the ERDF.

The third financial framework (2014–2017), during which environmental programmes received and continue to receive support under the Infrastructure and Environment Operational Programme in the 2014–2020 period (I&E OP).

The above programmes focused on the following priorities: water and wastewater management, improving the quality of surface water, waste management, protecting the Earth’s surface, improving air quality, counteracting environmental risks, nature conservation, and promoting pro-environmental behaviors [15,16,17].

The aim of this study was to analyze the dynamics of changes and spatial differences in the measures undertaken to protect and improve the quality of the environment, and to propose a set of indicators that support such assessments (the relevant data are publicly available in statistical databases). A Synthetic Indicator for Evaluating Environmental Performance (SIEEP) was developed and tested on Poland as an example. The proposed indicator can be modified for use in other countries by incorporating country-specific characteristics. Comprehensive programmes for analyzing and monitoring the effects of pro-environmental measures play an important role in environmental policy. Such tools should be developed with the use of the adequate variables (indicators). The indicators (variables) describing the progress made in various environmental protection categories were selected based on a review of the literature. A total of 27 variables divided into four categories were analyzed (refer to Table 1, Table 2, Table 3 and Table 4):

1. Water and wastewater management and water conservation.
2. Waste management and protection of the Earth’s surface.
3. Air pollution and climate control.
4. Nature conservation and promotion of pro-environmental behaviors.

## 2. Materials and Methods

### 2.1. Study Area

**Figure 1:**
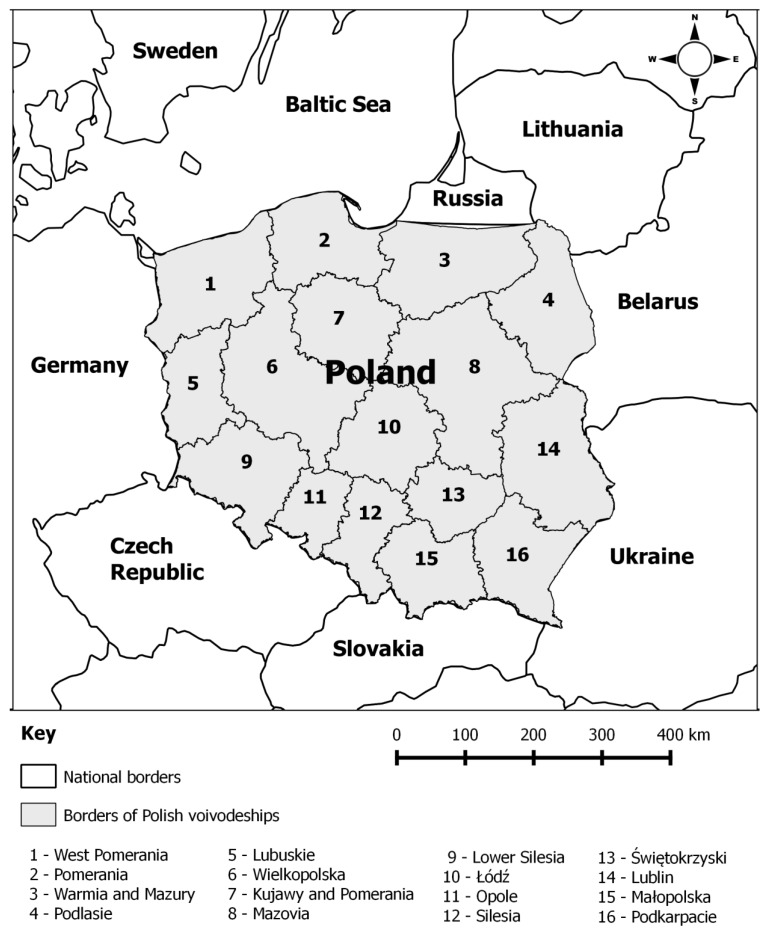
Map of Polish voivodeships and national borders.

Poland is situated in Central Europe (Figure 1). It borders Russia (Kaliningrad Region) and Lithuania in the north, and Belarus and Ukraine in the east. These borders (except the Polish-Lithuanian border) constitute the external borders of the European Union. In the south, the neighbouring countries are Slovakia and the Czech Republic. Germany is Poland’s western neighbor. The Baltic coast accounts for the major part of Poland’s northern border.

Poland has a total area of 312,695 km^2^ and a population of 38,411,000 (2018) [18]. Poland has 16 voivodeships divided into 380 counties (314 land counties and 65 cities with county rights). Counties are territorial units at Nomenclature of Territorial Units for Statistics (NUTS) 4 level. Counties are further subdivided into municipalities. Poland had 2478 municipalities in 2018 [18]. A total of 379 counties (314 land counties and 65 cities with county rights, which is equivalent to the number of counties in 2003) were analysed. Beginning from 2013, the data for Wałbrzych city with country rights and Wałbrzych county were aggregated because both counties were a part of the same territorial unit in the 2003–2012 period.

### 2.2. Methods

**Figure 2:**
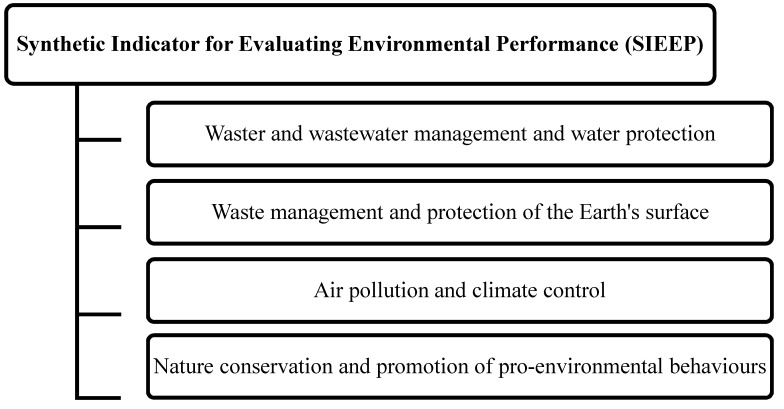
Structure of the Synthetic Indicator for Evaluating Environmental Performance (SIEEP).

The natural environment and environmental performance are highly complex concepts. For this reason, all activities aiming to improve the quality of the natural environment should be measured and monitored with the use of a broad set of indicators. Complex phenomena are analyzed with the use of synthetic variables, where a set of several indicators is replaced with a single synthetic indicator [19,20,21,22,23]. The priority axes of environmental protection programmes [15,16,17] were analyzed to develop a hierarchical structure for assessing the Synthetic Indicator for Evaluating Environmental Performance (SIEEP) and to identify four indirect criteria within the main criterion (SIEEP) (Figure 2). Detailed analyzes were based on indirect criteria, and the overall analysis was conducted based on the main criterion. 

The relevant data were analysed horizontally in four periods:

1. 2003 (base year—before Poland joined the European Union)
2. 2004–2006 (first financial framework)
3. 2007–2013 (second financial framework)
4. 2014–2017 (third financial framework—present situation).

The average values of selected variables were determined in the analyzed financial frameworks (2004–2006, 2007–2013, 2014–2017), and incremental values were analyzed based on data for the last year in a given time interval. Fixed prices in the base year (2003) were used to remove the effect of inflation on financial data. Data were acquired from the Local Data Bank for 2003–2017 developed by Statistics Poland.

A Synthetic Indicator for Evaluating Environmental Performance (SIEEP) was developed based on the Technique for Order Preference by Similarity to Ideal Solution (TOPSIS) method. In the applied method, a set of several indicators can be replaced with a single synthetic indicator. This method measures the Euclidean distance between the analyzed object (county) and the positive ideal solution (PIS), and the negative ideal solution (NIS). A smaller Euclidean distance denotes smaller differences in the diagnostic attributes of the evaluated objects, and, consequently, greater similarities between these objects in view of their diagnostic attributes [24]. The evaluated objects are ranked based on the value of the synthetic indicator of development. An object with the highest value of the synthetic indicator is characterized by the shortest distance from the PIS and the longest distance from the NIS [25,26,27]. This method is widely used in research [28,29,30,31,32] The following procedure was adopted to develop the synthetic indicator for indirect criteria and the main criterion (SIEEP):

1. The parameters (indicators) characterising the SIEEP were selected based on an analysis of its constituent elements (indirect criteria) in the literature [33,34,35,36,37,38].
2. The values of the analysed parameters (indicators) were standardised by zeroed unitarization, i.e., by converting destimulants into stimulants. This approach was adopted to ensure the comparability of the analyzed indicators. The following formulas were used [26]:
  (1) $$\mathrm{Stimulants}:\;z_{ik}=\frac{x_{ik}-\min_{i}\left\{x_{ik}\right\}}{\max_{i}\left\{x_{ik}\right\}-\min_{i}\left\{x_{ik}\right\}}$$
  (2) $$\mathrm{Destimulants}:\;z_{ik}=\frac{\max_{i}\left\{x_{ik}\right\}-x_{ik}}{\max_{i}\left\{x_{ik}\right\}-\min_{i}\left\{x_{ik}\right\}}$$
  where:
  *z*_ik_—standardized value of the *k*th parameter (indicator) for the *i*th object (county),
  *x*_ik_—real value of the *k*th parameter (indicator) for the *i*th object (county).
3. The coordinates of the positive ideal solution (A^+^) and the negative ideal solution (A−) were determined with the use of the following formulas:
  (3) $$A^{+}=\left(\underset{i}{\max}\left(z_{i1}\right),\underset{i}{\max}\left(z_{i2}\right),\;\ldots ,\;\underset{i}{\max}\left(z_{iK}\right))=(z_{1}^{+},z_{2}^{+},\ldots ,\;z_{K}^{+}\right)$$
  (4) $$A^{-}=\left(\underset{i}{\min}\left(z_{i1}\right),\underset{i}{\min}\left(z_{i2}\right),\;\ldots ,\;\underset{i}{\min}\left(z_{iK}\right))=(z_{1}^{-},z_{2}^{-},\ldots ,\;z_{K}^{-}\right)$$
  The ideal solutions take on the following form in zeroed unitarization:
  (5) $$z^{+}=\underset{K}{\underbrace{(1,\;1,\ldots ,1)\;z^{-}}}=\underset{K}{\underbrace{\left(0,\;0,\ldots ,0\right)}}$$
  where:
  *K*—number of parameters (indicators).
4. The Euclidean distance between the evaluated objects (counties), PIS z^+^ and NIS z− was calculated with the use of the below formulas:
  (6) $$d_{i}^{+}=\sqrt{\sum_{k=1}^{K}(z_{ik}-z_{k}^{+}}{)}^{2},d_{i}^{-}=\sqrt{\sum_{k=1}^{K}(z_{ik}-z_{k}^{-}}{)}^{2},\;i=1,\;2,\ldots ,\;N,\;k=1,\;2,\ldots ,\;K$$
  where:
  *N*—number of objects (counties).
5. The synthetic indicator (Si) for indirect criteria and the main criterion was calculated as follows [25]:
  (7) $$S_{i}=\frac{d_{i}^{-}}{d_{i}^{+}+d_{i}^{-}},\;\mathrm{where}\;0\;\leq \;\mathrm{Si}\;\leq \;1,\;\left(i\;=\;1,\;2,\;\ldots ,\;N\right)$$
6. The evaluated objects (counties) were arranged in a linear sequence, and the main criterion was divided into four typological classes based on the arithmetic mean and standard deviation of the synthetic indicator [23,26]:
  $S_{i}\geq \bar{S_{i}}+s_{S_{i}}$–class I–high value of the analysed parameter,
  $\bar{S_{i}}\leq S_{i}<\bar{S_{i}}+s_{S_{i}}$–class II–moderately high value of the analysed parameter,
  $\bar{S_{i}}-\;s_{S_{i}}\leq S_{i}<\bar{S_{i}}$–class III–moderately low value of the analysed parameter,
  $S_{i}<\bar{S_{i}}-s_{S_{i}}$–class IV–low value of the analysed parameter.
  where:
  *Si*—value of the synthetic indicator calculated for the main criterion with the TOPSIS method,
  $\bar{S_{i}}$—arithmetic mean of the synthetic indicator *Si*,
  $s_{S_{i}}$—standard deviation of the synthetic indicator *Si*.

The proposed classification was used to illustrate the spatial distribution of the analyzed phenomena.

## 3. Results

### 3.1. Indirect Criterion—Water and Wastewater Management, and Water Conservation

The average values of the indicators describing water and wastewater management in Poland (Table 1) point to a steady increase in the density of water supply and sewer networks in recent years. However, sewer networks are still not available in many Polish regions, particularly in rural areas. Many sewer networks are not connected to municipal wastewater processing plants. In 2003, the density of water supply networks was determined at 119.47 km∙km^−2^, and the density of sewer networks at 60.85 km∙km^−2^. In successive years, the greatest increase of more than 12% (to 143.26 km∙km^−2^) in the average density of water supply networks was observed in the 2007–2013 financial framework. In the analyzed periods, the density of sewer networks increased by nearly 40% (to 96.15 km∙km^−2^) relative to the preceding period. The expansion of water supply and sewer networks was slowed down in 2014–2017, and the density of water supply networks reached 151.10 km∙km^−2^, whereas the density of sewer networks reached 107.66 km∙km^−2^. It should also be noted that the average per capita spending on wastewater management and water conservation continued to increase until 2013 when it reached PLN 63.26 (Polish currency). In the third financial framework, however, the relevant expenditures decreased by 38% (to PLN 38.73) relative to the preceding years.

The observed increase in the values of the above variables was accompanied by a decrease in the average volume of treated municipal wastewater. This parameter was determined at 1659.26 dam^3^ (cubic decimeter)/100 km^2^ in 2003, and it continued to decrease by around 3% in each evaluated period to reach 1519.29 dam^3^/100 km^2^ on average in the third financing framework. In contrast, the average volume of treated industrial wastewater fluctuated across the analyzed periods. The volume of treated industrial wastewater reached 801.49 dam^3^/100 km^2^ in 2003, and a nearly 4% decrease was observed in the first financial framework. This parameter increased by more than 2% in the second financial framework and decreased by nearly 10% in the 2014–2017 period relative to the preceding period. A desirable increase was noted in the average throughput of wastewater treatment plants with enhanced removal of biogenic substances, which reached 0.12 m^3^∙day^−1^ per capita in 2003. This parameter increased by more than 13% in the 2004–2006 period, by more than 15% in 2007–2013, and by only 6.28% (to 0.16 m^3^∙day^−1^ per capita) in the 2014–2017 period. A steady increase in the size of wastewater treatment plants (PE) was also observed. In 2003, this parameter was determined at 1 PE, and it increased by 3% in the first financial framework, by 8.7% in the second financial framework, and by 9.8% in the third financial framework. The withdrawal of underground water in the industrial sector (in %) relative to total water consumption in the industry decreased in the analyzed periods, which is yet another positive phenomenon. The average value of this parameter increased by more than 4% between 2003 and 2004–2006, but a steady decrease was observed in successive periods. The average chemical oxygen demand (COD) of wastewater evacuated to water bodies or land continued to increase in the studied periods and was determined at 26,329.66 COD/100 km^2^ of county area in the third financial framework. The average biological oxygen demand (BOD_5_) of wastewater evacuated to water bodies or land fluctuated in the analyzed periods. This parameter decreased by 5% between 2003 and 2004–2006, increased by more than 3% in the 2007–2013 period, and decreased by more than 15% (to 3184.41 BOD_5_/100 km^2^ of county area) in the 2014–2017 period relative to the preceding financial framework. 

### 3.2. Indirect Criterion—Waste Management and Protection of The Earth’s Surface

The average per capita spending on waste management (Table 2) continued to increase in the analyzed periods. The relevant expenditure reached PLN 4.19 per capita in 2003, and it increased by 25% in the 2004–2006 period. In the second, longest financial framework (2007–2013), this parameter increased by more than 116%, but the most spectacular increase was noted in the 2014–2017 period, when municipal spending on waste management increased by more than 605% (to PLN 79.85, marking a nearly 145-fold increase from 2003) relative to the preceding period. The average per capita spending on waste collection in rural and urban areas varied considerably in the evaluated periods. This variable increased by more than 4% (to PLN 12.95) between 2003 and 2004–2006. In successive financial frameworks, waste collection costs increased by more than 25% (to PLN 16.19) in the 2007–2013 period and decreased by more than 6% (to PLN 15.17) in the 2014–2017 period.

Between 2003 and 2007–2013, the waste recycling rate (excluding municipal waste) increased from 69.19% to 79.27%. However, this parameter decreased nearly five-fold to only 16.82% in the 2014–2017 period. 

Waste minimization plays a very important role in waste management. Consumers should be educated to avoid buying products that generate hard-to-manage waste. At the same time, businesses should strive to produce packaging made of recyclable materials. However, eco-friendly strategies often increase the consumption expenditure of households to meet their everyday needs, including food. Waste production (excluding municipal waste) continued to decrease steadily in the evaluated periods. This parameter was determined at 129,180 mg/100 km^2^ in 2003, and it increased by only 0.53% in the 2004–2006 period. A nearly 10% decrease was noted in the 2007–2013 period, followed by a decrease of 6.13% in the 2014–2017 period.

Landfilling is the least environmentally sustainable waste management strategy. Defunct landfill sites are highly degraded areas. Considerable public funds are allocated to landfill reclamation and reducing the area of non-reclaimed landfills. In 2003, the average area of non-reclaimed land was determined at 11.61 ha/100 km^2^. This parameter decreased by nearly 5% between 2004 and 2006, by more than 15% between 2007 and 2013, and by nearly 9% (to 8.62 ha/100 km^2^) between 2014 and 2017. As landfill sites were closed or abandoned, the amount of landfilled waste was reduced from 1,936,060 mg/100 km^2^ in 2003 by around 8% in the first and second financial framework, and by more than 5% in the third financial framework.

### 3.3. Indirect Criterion—Air Polution and Climate Control

Municipal spending on air pollution and climate control increased steadily in the analyzed financial frameworks (Table 3). The relevant expenditures increased by 213% from PLN 3242.34/10,000 population on average in 2003 to PLN 10,152.80/10,000 population between 2004 and 2006. This parameter increased by 50% in the 2017–2013 period, whereas a five-fold increase to PLN 77,651.17/10,000 population on average was noted in the 2014–2017 period.

Growing investment spending contributes to the implementation of systems and devices for combatting air pollution. The efficiency of the installed equipment for reducing particulate and gaseous pollution decreased in the analyzed financial frameworks. In 2003, the average efficiency of air pollution control devices in Polish counties was determined at 654.61 mg/year, and it increased by more than 44% between 2004 and 2006. In the following financial framework, however, this parameter decreased by 87% to only 123 mg/year. In the last analyzed period, equipment efficiency increased 2.5-fold to 314.73 mg/year.

In 2003, the average density of gas grids was 81.92 km∙km^−2^. This parameter increased by only 4% between 2004 and 2006, by more than 10% in the 2007–2013 period, and by 7% between 2014 and 2017.

Particulate and gaseous emissions from hazardous industrial plants continued to decrease steadily in the studied periods. In 2003, particulate emissions in counties were determined at 1.77 mg/year/1 km^2^ on average. This parameter continued to decrease in successive years to reach 0.48 mg/year/1 km^2^ in the 2014–2017 period, marking a four-fold decrease from the base year. Between 2004 and 2006, gaseous emissions increased by nearly 2.5% from 2561.26 mg/year/1 km^2^ in 2003. This parameter decreased gradually in the following financial frameworks: by 3% in the 2007–2013 period, and by more than 4% between 2014 and 2017.

### 3.4. Indirect Criterion—Nature Conservation and Promotion of Pro-Environmental Behaviours

The number of households connected to public sewers increased steadily in the analyzed periods (Table 4). The percentage of the resident population connected to a public sewer system increased from more than 52% in 2003 to nearly 67% in the third financial framework. 

In 2003, the proportion of protected areas in Poland’s total area reached nearly 29%. A decrease to 28% was noted in 2013, followed by an increase to the base year value (2003) between 2014 and 2017. 

The analyzed periods were characterized by a steady increase in forest area. The average share of forest area in county area was determined at less than 5% in 2003, and it increased by nearly 2% between 2004 and 2006 (but did not exceed the 5% mark). In the 2007–2013 period, this parameter increased by nearly 5.5% relative to the preceding period. In the last financial framework, more than a 4% increase in forest area was observed relative to previous period. 

The average per capita spending on public green spaces was PLN 5.03 in 2003, and it continued to increase in successive periods by 17%, 66%, and 26%, respectively. Water and natural gas consumption continued to decrease in the analyzed time series. The average per capita consumption of mains water decreased from 35.62 m^3^ in 2003 by around 3% in 2004–2006, by around 2% in the 2007–2013 period, and by more than 2% in the 2014–2017 period. Per capita consumption of natural gas was determined at 469.22 m^3^ in 2003, and the corresponding decrease was significantly higher, in particular in the first and second financial framework (26% and 27%, respectively). The discussed parameter decreased by more than 8% in 2014–2017. 

### 3.5. Main criterion—Synthetic Indicator for Evaluating Environmental Performance (SIEEP)

**Figure 3:**
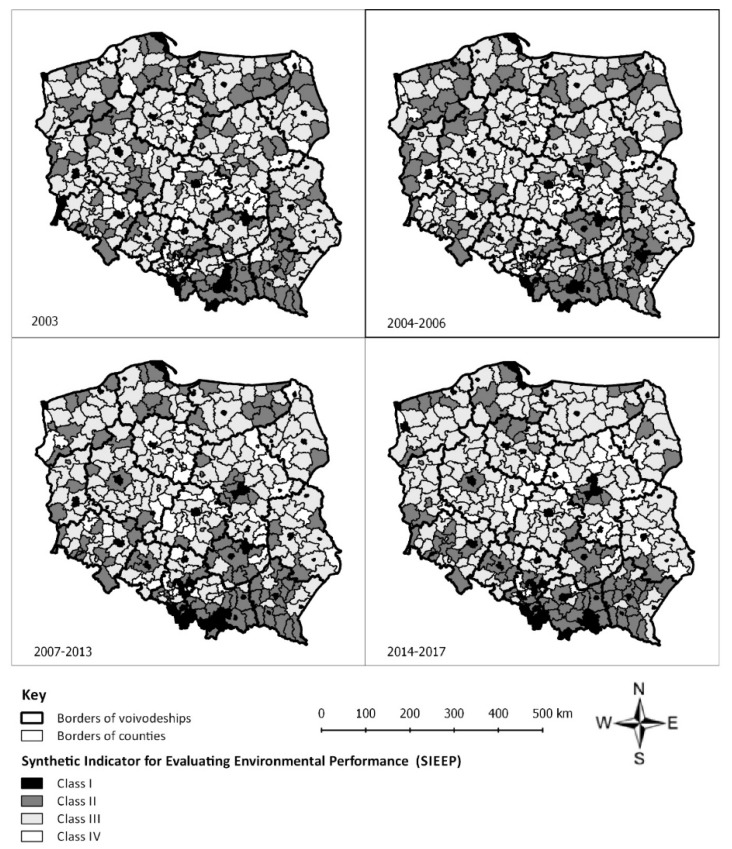
Spatial distribution of Polish counties divided into four classes based on the values of the Synthetic Indicator for Evaluating Environmental Performance (SIEEP).

**Figure 4:**
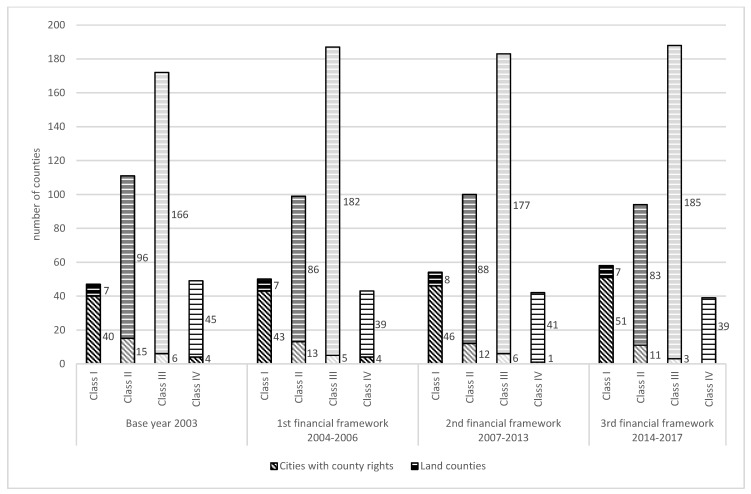
Classification of Polish counties in the analyzed periods.

**Figure 5:**
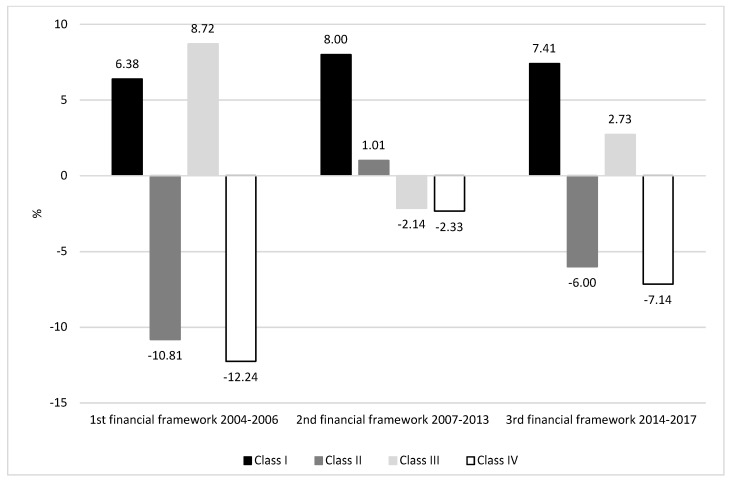
Dynamics of changes in the analyzed financial frameworks.

The collected data (Table 1, Table 2, Table 3 and Table 4) were analyzed to rank Polish counties based on the value of the SIEEP. The spatial distribution of the evaluated parameters is presented in Figure 3. The classification of Polish counties in the evaluated periods is presented in Figure 4, and the dynamics of changes is presented in Figure 5.

At the beginning of the analyzed period (2003), the highest indicator values (class I) were noted in 12% of Polish counties. Nearly 30% of counties belonged to class II. The highest proportion of counties (more than 45%) belonged to class III. The lowest values of the synthetic indicator (class IV) were noted in nearly 13% of counties. The spatial distribution of classes varied considerably. Class I was composed mainly of cities with county rights. Class II counties were clustered mainly in southern Poland in the voivodeships of Małopolska and Świętokrzyskie, and partly in northern and north-eastern Poland. Class IV counties were situated mainly in the voivodeships of Łódź (9 out of 24 counties in that voivodeship) and Kujawy and Pomerania (6 out 23 counties in that voivodeship).

The value of the synthetic indicator remained fairly constant in 301 counties in the first financial framework. The synthetic indicator increased in 39 counties and decreased (by one class) in 39 counties. The most noticeable changes were observed in class II counties whose number increased in north-western Poland and decreased in north-eastern Poland, and in the voivodeships of Wielkopolska, and Kujawy and Pomerania. Public spending on air pollution and climate control increased more than three-fold between 2003 and the first financial framework, whereas the performance of the equipment for absorbing particulate and gaseous pollutants increased nearly 1.5-fold in the corresponding period. Natural gas consumption decreased by more than 25%, and waste management expenditure began to increase. 

The most profound changes in the analyzed variables were noted in the second financial framework (2007–2013). The number of class I counties increased by 8%, and the number of class II countries increased by more than 1%. The number of class III counties decreased by more than 2%, and the number of class IV counties decreased by 2.3% relative to the preceding period. Between 2007 and 2013, 280 counties did not change their ranking, 45 counties were degraded to a lower class, and 54% counties were promoted to a higher class, including the city of Katowice with county rights which moved up two classes (from class III to class I). Class II counties were clustered in southern Poland, and the number of class II counties continued to decrease in the northern parts of the country. Sewer network density increased, and waste management expenditure more than doubled in the 2007–2013 period. Waste disposal expenditure increased by 25% in rural and urban areas, and an increase was also observed in spending on air pollution and climate control and the maintenance of green spaces. Natural gas consumption increased. The performance of the equipment for capturing particulate and gaseous pollutants decreased in the analyzed period. A decrease was also noted in particulate emissions from hazardous industrial plants. 

Further positive changes were also noted in the last financial framework (2014–2017). In comparison with the preceding period, the highest, nearly 7.5% increase was observed in the number of class I counties. The number of class II counties decreased by 6% relative to 2007–2013. Similarly, to the remaining periods, class III was represented by the highest number of counties (nearly 50% of Polish counties) which increased by nearly 3% in the 2014–2017 period. The smallest number of counties belonged to class IV which witnessed a further decrease of more than 7% in the last financial framework. None of class IV counties were cities with county rights. 

In the third financial framework (2014–2017), 267 counties did not change their status from the previous period. The value of the synthetic indicator increased in 58 counties, and Kraków city with county rights was promoted from class III to class I. In this period, 54 counties regressed by one class in the ranking. The number of class II counties continued to increase in southern Poland and decrease in the northern and eastern parts of the country. Public spending on wastewater management and water conservation decreased considerably, and a substantial decrease was also observed in the waste recycling rate (excluding municipal waste). Particulate matter emissions from hazardous industrial plants decreased. Waste management expenditure increased seven-fold relative to the preceding period and 20-fold relative to 2003. Public spending on air pollution and climate control increased five-fold, and the performance of the equipment for absorbing particulate and gaseous pollutants increased more than 2.5-fold. More funds were also channelled to the maintenance of public green spaces. 

## 4. Discussion

### 4.1. Water and Wastewater Management and Water Conservation

Water is the most ubiquitous chemical compound and the main component of all living organisms on Earth. This essential abiotic resource plays a particularly important role in the functioning of ecosystems. Water is a renewable resource, and its availability fluctuates over time. Water is also vital to the economy, which is why it should be protected against pollution and rationally managed. Qualitative and quantitative protection of water resources is an integral element of environmental protection activities [39,40]. In Poland, surface water resources are estimated at 62 km^3^, ranging from less than 40 km^3^ in very dry years to more than 90 km^3^ in very wet years. Ground water resources are replenished at a slower rate, and they are estimated at 16 km^3^ in Poland. Water resources per capita are determined at 1580 m^3^/year in Poland, which is three times less than the European average [41]. In recent years, the absence of snow cover in winter has significantly compromised the availability of surface and ground water. These problems need to be urgently addressed by building water retention facilities and monitoring the quality and quantity of water resources.

Poland is characterised by adverse climatic and hydrological conditions, and its underground water resources are very low relative to other European countries [42]. The withdrawal of underground water in the industrial sector relative to total water consumption in industry increased from 74.21% in 2003 to 77.73% in the first financial framework. A small but steady decrease in this parameter was observed in the second and third financial framework. 

Water management and the associated infrastructure, including water supply and sewer networks, represent the priority areas of the EU’s environmental policy. The framework for the Community action in the field of water policy was established by the EU Framework Water Directive [43,44]. The availability of water supply and sewer networks continued to increase steadily in Poland in the analyzed periods. In the third financial framework, the density of the water distribution network increased by 26.5% and the density of the sewer network increased by 76.9% from 2003. However, many locations, in particular in rural areas, still lack access to public sewers and wastewater treatment plants. The results of the analysis also revealed positive changes. In the last financial framework, the size of municipal wastewater plants (PE) increased by 23%, and the performance of wastewater treatment plants with enhanced removal of biogenic pollutants increased by 33% relative to 2003.

In effective water and wastewater management, mainly in rural areas, contributes to environmental pollution in Poland. The volume of treated industrial wastewater tended to fluctuate in the analyzed periods. A minor 2% increase in the volume of treated wastewater was observed between the second and the third financial framework. However, this parameter decreased by around 11% between 2003 and the 2014–2017 period. These differences could be attributed to fluctuations in business cycles, seasonal production in selected market segments [45], and the implementation of water-saving technologies [46].

The volume of treated municipal waste decreased steadily by around 3% in the analyzed periods, which can be attributed to lower water consumption (indirect criterion – nature conservation and promotion of pro-environmental behaviours) and a decrease in wastewater generation. The volume of untreated wastewater evacuated to the environment decreased steadily in the evaluated periods from 164,668.4 dam^3^ in 2003 to 128,205.4 dam^3^ in the 2004–2006 period, 51,635.1 dam^3^ in the 2007–2013 period, and 1945.0 dam^3^ in the 2014–2017 period [18].

New wastewater treatment sites were built, and the existing facilities were upgraded under the National Municipal Wastewater Treatment Program (NMWTP) [47]. The NMWTP decreased the volume of untreated wastewater and reduced pollutant loads in treated effluents discharged to the environment [48]. Chemical oxygen demand (COD) and biochemical oxygen demand (BOD_5_) are the key indicators of water and wastewater quality. Chemical oxygen demand increased from 3% to 5%, whereas BOD_5_ fluctuated in the analyzed periods. An approximately 3% increase in BOD_5_ was noted between the second and third financial framework. However, the overall pollutant loads decreased by nearly 17% between 2003 and the last financial framework, which is a highly satisfactory result. 

In Poland, water and wastewater management projects are financed mainly by municipalities which are responsible for building and maintaining the relevant infrastructure [49]. The relevant expenditures are planned in municipal budgets each year. Municipal spending on water and wastewater management increased in the first and second financial framework (by approx. 20% relative to the preceding period), whereas a considerable decrease was noted in the third financial framework (from PLN 44.61 per capita in 2003 to PLN 38.73 per capita between 2014 and 2017). In the third financial framework, funds for water and wastewater management projects will be allocated until the end of 2020; therefore, the average expenditure in this period is likely to increase. 

The water policy has to be revised in all stages of the planning process to promote the development of integrated water and wastewater management systems. The relevant measures are being implemented in Poland and other countries in the process of harmonizing national laws with the provisions of the Framework Water Directive and the Floods Directive [50].

### 4.2. Waste Management and The Protection of The Earth’s Surface

Waste generation and management are also key aspects of environmental protection. Waste poses health risks for humans and animals, and it contributes to water, air, soil and land pollution. Waste exerts a negative impact on plants and agricultural crops. Ineffective waste management compromises the quality of natural resources and the local landscape [51].

The importance of waste management and the protection of the Earth’s surface is increasingly recognised in Poland, as demonstrated by a steady and significant increase in public spending on waste management in the last financial framework. Municipal expenditure on waste management increased nearly 20-fold from PLN 4.19 per capita in 2003 to PLN 79.85 per capita between 2014 and 2017. In the corresponding period, spending on waste collection in rural and urban areas increased at a far slower rate, whereas a decrease of around 6% in this parameter was noted between the 2007–2013 period and the 2014–2017 period. 

Waste generation is inextricably linked with the development of civilisation, and the volume of produced waste is largely determined by economic growth and the magnitude and patterns of consumption [52]. Despite steady GDP growth, annual waste generation (excluding municipal waste) continued to decrease in the analyzed periods, with the exception of a minor 0.5% increase between 2003 and the 2004–2006 period. The evaluated parameter decreased by approximately 14% between 2003 and the last financial framework. 

Waste management poses a considerable problem in an era when global efforts are being made to increase resource efficiency, minimize the negative impact of waste on the environment, and reduce landfill waste [53]. Defunct landfills have to be reclaimed to prevent toxic substances from reaching surface and underground water as well as ambient air. Reclaimed landfills have to be integrated with the surrounding environment [51]. Binding technical requirements for the reclamation of waste and landfills have been introduced in Polish and EU regulations. The area of non-reclaimed landfills has decreased steadily in the analyzed periods. A nearly 26% decrease was observed between 2003 and the third financial framework. The volume of landfilled waste also decreased by 20% in the corresponding period. 

The long-term goal of waste management is to reduce waste landfilling and increase the reuse and recycling of the main waste streams, including municipal waste and packaging waste. In recent years, a product policy framework that contributes to a circular economy has been proposed by the Zero Waste Europe movement. Efforts are being made to reduce waste generation to a minimum. The zero waste concept postulates that waste is a potential resource with economic value rather than a problem to be dealt with [54,55,56].

The recycling rate is a measure of the efficiency of waste management policies. There is a general scarcity of modern waste management systems in Poland, and Polish consumers are reluctant to sort waste. According to the 2018 report of the Supreme Audit Office, Poland has one of the lowest recycling rates in the EU [57]. The recycling rate (excluding municipal waste) increased by 5.7% between 2003 and the 2004–2006 period, and by 8.3% between the 2004–2006 period and 2007–2013 period. However, a nearly five-fold decrease in the recycling rate was noted in successive periods (79.27% in 2007–2013, and 16.82% in 2014–2017), which gives serious cause for concern. 

Education and awareness-building play a very important role in waste management. Free brochures and pamphlets distributed by municipal waste management services and the local authorities are more effective at raising public awareness on waste than Internet sources. Polish consumers are becoming increasingly aware that adequate waste management contributes to environmental protection. The observed improvement can be attributed mainly to the success of campaigns promoting waste reduction, reuse and recycling [58].

### 4.3. Air Pollution and Climate Control

Economic growth contributes to environmental degradation. Air pollution is one of the most frequently discussed topics in the global environmental debate. Air pollution is a mixture of solid, liquid, and gaseous substances, as well as electromagnetic radiation that can reach harmful concentrations in ambient air [59].

Particulate matter emissions from hazardous industrial plants decreased steadily in the analyzed periods. This parameter decreased more than three-fold from 1.77 mg/year/km^2^ in 2003 to 0.48 mg/year/km^2^ in 2014–2017. A steady improvement was also observed in gaseous pollutant emissions which decreased by 5% between 2003 and the 2014–2017 period (excluding the first financial framework when the analyzed parameter increased by around 3% relative to the base year value). Significant changes in gaseous and particulate air pollution have not been observed in Poland because bituminous coal continues to be the most important primary fossil fuel in the national economy. In 2016, bituminous coal accounted for 39.8% of primary energy sources in Poland. The consumption of bituminous coal and biomass is also high in households. Around 46% of the energy consumed by Polish households was generated from bituminous coal and biomass [14].

Despite the above, certain progress has been made in air pollution and climate control over the past two decades. The failure to address environmental issues in the past has contributed to serious and costly problems, and many Polish cities suffer from the highest air pollution levels in Europe [60]. For this reason, Polish voivodeships that are most affected by air pollution (Małopolska, Silesia, Opole, Mazovia, Łódź, Lower Silesia, Podkarpacie, and Wielkopolska) have adopted anti-smog regulations to reduce pollutant emissions to ambient air. In those voivodeships, the use of boilers, furnaces and fireplaces has been restricted or prohibited, and low-quality fossil fuels have been banned in the most affected areas [14].

The main burden of planning and implementing air pollution control policies rests on voivodeship authorities which develop air protection programs (APPs). These programs list protective measures that have to be implemented by municipal authorities in the areas covered by APPs. Municipalities introduce the preventive and remedial actions set forth by APPs [61]. Municipal expenditure on systems and solutions for air pollution and climate control continued to increase in the analyzed periods. The relevant spending increased nearly 24-fold from PLN 3242.44/10,000 population in 2003 to PLN 77,651/10,000 population in the third financial framework. 

Air pollution control is no longer the sole domain of public administration. The introduction of stringent air quality standards also forces businesses to adopt environmentally friendly solutions. The number of green business that actively strive to minimize their impact on the environment is on the rise. Sustainable businesses introduce eco-friendly technologies and production systems to reduce emissions of particulate matter and gaseous pollutants.

Growing investment spending contributes to the implementation of systems and devices for combatting air pollution. The efficiency of the installed equipment for reducing particulate and gaseous pollution fluctuated considerably in the analyzed financial frameworks. A two-fold decrease in the above parameter was observed between 2003 (654.61 mg/year) and 2014–2017 (314.73 mg/year), mainly due to a reduction in pollutant emissions [62] resulting from the implementation of Best Available Techniques (BAT) in industry and the corresponding legal solutions (integrated environmental permits). It should also be noted that hazardous industrial plants are gradually losing their competitive edge, and their number continues to decrease in Poland. However, despite considerable reductions in industrial emissions, air quality standards are still not met in Poland [63].

In Poland, bituminous and brown coal is the main source of air pollution, which increases the popularity of alternative fuels, including natural gas. The growing demand for natural gas stimulates infrastructure development. The density of the gas grid increased at a rate of 4–10% between the analyzed periods. However, a comparison of the data for 2003 and 2014–2017 revealed more than a 23% increase in gas grid density between the base year and the third financial framework.

In Poland, air pollution and climate change are addressed by legal regulations and planning documents at all levels of government. Due to the multitude of documents on air quality control, attempts were made to generate a single policy document describing current levels of air pollution. In 2015, the Ministry of the Environment adopted the National Air Pollution Control Program until 2020 (with a long-term horizon until 2030), which aims to improve the quality of air throughout Poland [63].

### 4.4. Nature Conservation and Promotion of Pro-Environmental Behaviours

Poland is characterised by considerable biological diversity, both in terms of the number of species and environmental values. This diversity is shaped by a relatively large area of forests and wetlands as well as extensive agriculture. Conservation of biological diversity guarantees the stability of ecosystem functioning and the maintenance of ecological balance. In an ongoing effort to preserve its environmental values, Poland has been implementing various types of nature conservation measures to protect natural areas, objects, plant and animal species, and their habitats [64]. In 2017, protected areas spanned 10,175,600.82 hectares and accounted for nearly 33% of Poland’s territory. Only minor differences in the size of protected areas were observed in the analyzed periods. Between the base year and the third financial framework, the proportion of protected areas decreased by approximately 1%.

Forests are natural formations that significantly contribute to the preservation of ecological balance and biological and landscape diversity. Forests play numerous protective roles by regulating the natural water cycle, preventing floods, and landslides, protecting soil and landscapes against erosion and desertification, and reducing pollution. Forests are also a form of land use that enhances the production of various biological commodities. Forests have considerable market value, and they constitute a public good that influences the quality of life [65]. In 2017, Polish forests occupied an area of 9,446,962.37 ha and accounted for 29.6% of the national territory. Most Polish forests are owned by the State Treasury. Only 20% of forests are owned by municipalities and individuals [18]. The proportion of private and municipal forests in total forest area continued to increase during the evaluated periods. This parameter increased by more than 12% between 2003 and the 2014–2017 period. Subsidies for the afforestation of low-quality farmland granted under rural development programs have undoubtedly contributed to the observed increase in forest area.

Greening projects also play an important role in nature conservation [66]. Open green spaces in densely developed cities and villages play aesthetic, recreational, protective and health-promoting roles. They improve the quality of life and contribute to the well-being of local communities. In 2017, open green spaces occupied an area of 94,629.18 ha, accounting for 0.3% of Poland’s territory. Most public green spaces were situated in urban areas and accounted for 3.3% of the area of Polish cities [18]. A steady expansion of green spaces and an increase in municipal spending on the maintenance of public greenery were noted in the analyzed periods. The highest 66% increase was observed between the first and the second financial framework. Public expenditure on the maintenance of open green spaces increased 2.5-fold between 2003 and the 2014–2017 period. 

Environmental awareness plays a very important role in nature conservation. Environmental awareness is a broad term that encompasses environmental knowledge, recognition of natural phenomena, the existing correlations, causes and effects, and the willingness to participate in wildlife conservation based on an analysis of environmental costs and benefits [67,68]. Pro-environmental attitudes are manifested by rational consumer behaviours that aim to minimize the use of natural resources, such as water and gas. Water consumption decreased steadily by 2–3% between the analyzed periods, and the noted decrease reached nearly 7% between the base year and the third financial framework. Water metering contributes to rational resource use by reducing water wastage. Water-tight plumbing and pro-environmental behaviours also play an important role in resource conservation [48]. Natural gas consumption also decreased between the analyzed periods (despite the observed increase in the density of the gas grid, refer to Table 3). This parameter decreased more than two-fold between the base year and the third financial framework. Households use gas mainly for indoor heating, water heating and cooking [69]. The observed decrease in gas consumption was influenced by lower water use, as well as the growing popularity of alternative heating systems and cooking appliances (such as electric and microwave ovens). Thermal insulation for buildings, thermostatic valves, and a reduction in heating degree days also contributed to lower gas use [70].

The popularity of integrated systems including water distribution pipelines, sewers, and wastewater treatment plants is on the rise due to growing levels of environmental awareness. Wastewater treatment plants not only process sewage, but also recover heat, water, fuels, and nutrients for secondary use [71]. The number of wastewater treatment plants increased by 18% from 2761 in 2003 to 3258 in 2017 [18]. This increase can be attributed mainly to higher spending on water and wastewater infrastructure. As a result, the proportion of households connected to sanitary sewers increased by 27% between the base year and the third financial framework. Wastewater treatment systems are also evolving, and mechanical treatment devices are being replaced with high-performance systems that are more effective in removing nitrogen and phosphorus compounds from the effluent [64].

The past three decades witnessed a clear increase in environmental awareness levels. These changes have contributed to a rise in initiatives aiming to minimise the negative impact of human activities on the environment, secure the natural environment for future generations, and promote a holistic approach to environmental protection [14].

### 4.5. Synthetic Indicator for Evaluating Environmental Performance (SIEEP)

Comprehensive systems for analysing and monitoring the effectiveness of environmental protection activities are essential for achieving environmental sustainability. Such systems facilitate environmental management and policies at all stages of the decision-making process [72,73]. In many cases, environmental changes are analyzed separately for every parameter without addressing the complex nature of the relevant problems [73,74]. This approach prevents effective allocation of public funds for environmental protection. The applicability and reliability of environmental analyses can be substantially improved by incorporating composite, aggregate indicators in assessments of environmental pressures. To guarantee their effective use, environmental indicators should be aggregated not only in terms of their thematic scope, but also their spatial distribution [75]. Synthetic indicators play a significant role in environmental analyses by presenting complex data from numerous sources in a simplified and practical manner [76].

Environmental indicators facilitate environmental monitoring and reporting [73], and they have been adopted by numerous institutions and organisations [77]. This study evaluated the dynamics of changes and spatial differences in environmental protection activities, and the results of the analysis were used to develop the SIEEP based on a set of variables that support such assessments. The proposed synthetic indicator was tested on the example of Poland, a Member State of the EU. The analyzed variables are published by statistical organisations and constitute public domain data (in Poland, statistical data are collected and published by Statistics Poland). The SIEEP is a universal indicator that can be applied in other countries in view of their specific characteristics. The generated information about environmental changes and processes is highly useful in policy-making and environmental management.

The analyzed variables affect the value of the synthetic indicator and the classification of the studied entities. The quality of diagnostic variables largely determines the reliability of classification results, the formulated conclusions and the decision-making process [78,79]. The main limitation of this study was the limited availability of indicators describing environmental protection activities undertaken in territorial units at the NUTS 4 level across years. Therefore, further research is needed to develop methods and tools for detailed analyses where indicators are assigned weights and are divided into categories based on their availability.

## 5. Conclusions

The implementation of publicly funded environmental protection activities in Poland minimizes the negative impact of human activities on the environment. Positive changes in the average values of the analyzed variables and the improved performance of local administrative units indicate that environmental expenditures in successive financial frameworks have contributed to an improvement in the quality of the natural environment. The number of class I and III counties increased, whereas a minor decrease was observed in the number of class II counties. Most importantly, a steady decrease was noted in the number of class IV counties with the lowest values of the analyzed indicator.

The proposed synthetic indicator serves an important analytical function by facilitating the identification and monitoring of environmental processes in an organised, but flexible manner. The indicator can be used to develop the appropriate metrics for conducting reliable environmental assessments and developing effective and cohesive environmental reports. The described methodology can be deployed to develop comprehensive systems for monitoring the effectiveness of environmental protection activities at the level of territorial units.

## Figures and Tables

**Table 1:** Selected indicators for analysing the indirect criterion—water and wastewater management and water conservation, and average indicator values for Poland.

No.	Indicator	Average Values for Poland			
		Base Year	1st Financial Framework	2nd Financial Framework	3rd Financial Framework
		2003	2004–2006	2007–2013	2014–2017
1	Density of water supply networks, km∙km^−2^—S	119.47	127.28	143.26	151.10
2	Density of sewer networks, km∙km^−2^—S	60.85	69.53	96.15	107.66
3	Municipal spending per capita on wastewater management and water conservation (PLN) *—S	44.61	53.23	63.26	38.73
4	Size of wastewater treatment plants (PE)—S	1.00	1.03	1.12	1.23
5	Treated municipal wastewater per 100 km^2^ (dam^3^)—S	1659.26	1614.13	1563.48	1519.29
6	Treated industrial wastewater per 100 km^2^ (dam^3^)—S	801.49	769.60	787.43	714.44
7	Throughput of wastewater treatment plants with enhanced removal of biogenic impurities per capita (m^3^∙day^−1^)—S	0.12	0.13	0.15	0.16
8	Withdrawal of underground water in the industrial sector relative to total water consumption in industry (%)—D	74.21	77.73	77.54	73.39
9	BOD_5_ load of wastewater evacuated to water bodies or land per 100 km^2^—D	3833.42	3635.80	3748.12	3184.41
10	COD load of wastewater evacuated to water bodies or land per 100 km^2^—D	23,492.61	24,186.74	25,318.03	26,329.66
USD 1 * EUR 1 *		PLN 3.8889 PLN 4.3978	PLN 3.3304 PLN 4.1515	PLN 2.9556 PLN 4.0177	PLN 3.6615 PLN 4.2473

* Average exchange rate quoted by the National Bank of Poland (accessed on 14 June 2019). S—stimulant, D—destimulant, PLN—Polish currency, PE—population equivalent, dam^3^—cubic decimetre, BOD_5_—biological oxygen demand, COD—chemical oxygen demand. Source: own elaboration based on Statistics Poland data.

**Table 2:** Selected indicators for analysing the indirect criterion—waste management and protection of the Earth’s surface, and average indicator values for Poland.

No.	Indicator	Average Values for Poland			
		Base Year	1st Financial Framework	2nd Financial Framework	3rd Financial Framework
		2003	2004–2006	2007–2013	2014–2017
1	Municipal spending per capita on waste management (PLN) *—S	4.19	5.24	11.31	79.85
2	Municipal spending per capita on waste collection (PLN) *—S	12.40	12.95	16.19	15.17
3	Annual waste production (excluding municipal waste) in ‘000 mg per 100 km^2^—D	129.18	129.87	117.90	110.67
4	Landfilled waste in ‘000 mg per 100 km^2^ (landfills, disposal sites for mining waste, such as spoil tips and sediment ponds)—D	1936.06	1774.15	1632.06	1548.42
5	Waste recycling rate (excluding municipal waste) (%)—S	69.19	73.19	79.27	16.82
6	Non-reclaimed landfills in ha per 100 km^2^—D	11.61	11.08	9.47	8.62
USD 1 * EUR 1 *		PLN 3.8889 PLN 4.3978	PLN 3.3304 PLN 4.1515	PLN 2.9556 PLN 4.0177	PLN 3.6615 PLN 4.2473

* Average exchange rate quoted by the National Bank of Poland (accessed on 14 June 2019). Source: own elaboration based on Statistics Poland data.

**Table 3:** Selected indicators for analyzing the indirect criterion—air pollution and climate control, and average indicator values for Poland.

No.	Indicator	Average Values for Poland			
		Base Year	1st Financial Framework	2nd Financial Framework	3rd Financial Framework
		2003	2004–2006	2007–2013	2014–2017
1	Municipal spending on air pollution and climate control per 10,000 population (PLN) *—S	3242.34	10152.80	15171.74	77651.17
2	Density of the gas grid, km∙km^−2^—S	81.92	85.37	94.11	100.92
3	Reduction in particulate and gaseous pollution (mg∙year^−1^)—S	654.61	944.88	122.95	314.73
4	Particulate emissions from hazardous industrial plants per 1 km^2^ (mg∙year^−1^)—D	1.77	1.43	0.76	0.48
5	Gaseous emissions from hazardous industrial plants per 1 km^2^ (mg∙year^−1^)—D	2561.26	2625.02	2544.65	2433.37
USD 1 * EUR 1 *		PLN 3.8889 PLN 4.3978	PLN 3.3304 PLN 4.1515	PLN 2.9556 PLN 4.0177	PLN 3.6615 PLN 4.2473

* Average exchange rate quoted by the National Bank of Poland (accessed on 14 June 2019). Source: own elaboration based on Statistics Poland data.

**Table 4:** Selected indicators for analyzing the indirect criterion—nature conservation and promotion of pro-environmental behaviors, and average indicator values for Poland.

No.	Indicator	Average Values for Poland			
		Base Year	1st Financial Framework	2nd Financial Framework	3rd Financial Framework
		2003	2004–2006	2007–2013	2014–2017
1	Per capita consumption of mains water in m^3^—D	35.62	34.60	34.00	33.23
2	Per capita consumption of natural gas in m^3^—D	469.22	349.20	254.96	233.86
3	Proportion of forests (private and municipal) in total area (%)—S	4.89	4.98	5.25	5.48
4	Municipal spending per capita on public green areas (PLN) *—S	5.03	5.89	9.78	12.37
5	Percentage of total population connected to a public sewer system (%)—S	52.34	54.95	60.30	66.48
6	Proportion of protected areas in total area (%)—S	28.62	28.13	28.04	28.25
USD 1 * EUR 1 *		PLN 3.8889 PLN 4.3978	PLN 3.3304 PLN 4.1515	PLN 2.9556 PLN 4.0177	PLN 3.6615 PLN 4.2473

* Average exchange rate quoted by the National Bank of Poland (accessed on 14 June 2019). Source: own elaboration based on Statistics Poland data.
